# Long-Term Prognosis of Patients with Carbon Monoxide Poisoning: A Nationwide Cohort Study

**DOI:** 10.1371/journal.pone.0105503

**Published:** 2014-08-28

**Authors:** Chien-Cheng Huang, Min-Hsien Chung, Shih-Feng Weng, Chih-Chiang Chien, Shio-Jean Lin, Hung-Jung Lin, How-Ran Guo, Shih-Bin Su, Chien-Chin Hsu, Chi-Wen Juan

**Affiliations:** 1 Department of Emergency Medicine, Chi-Mei Medical Center, Tainan, Taiwan; 2 Department of Child Care and Education, Southern Taiwan University of Science and Technology, Tainan, Taiwan; 3 Department of Environmental and Occupational Health, College of Medicine, National Cheng Kung University, Tainan, Taiwan; 4 Department of Emergency Medicine, Kuo General Hospital, Tainan, Taiwan; 5 Department of Emergency Medicine, Chi-Mei Medical Center, Liouying, Tainan, Taiwan; 6 Departments of Medical Research, Chi-Mei Medical Center, Tainan, Taiwan; 7 Department of Nephrology, Chi-Mei Medical Center, Tainan, Taiwan; 8 Department of Food Nutrition, Chung Hwa University of Medical Technology, Tainan, Taiwan; 9 Department of Pediatrics, Chi-Mei Medical Center, Tainan, Taiwan; 10 Department of Emergency Medicine, Taipei Medical University, Taipei, Taiwan; 11 Department of Biotechnology, Southern Taiwan University of Science and Technology, Tainan, Taiwan; 12 Department of Occupational and Environmental Medicine, National Cheng Kung University Hospital, Tainan, Taiwan; 13 Department of Occupational Medicine, Chi-Mei Medical Center, Tainan, Taiwan; 14 Department of Leisure, Recreation and Tourism Management, Southern Taiwan University of Science and Technology, Tainan, Taiwan; 15 Department of Medical Research, Chi Mei Medical Center, Liouying, Tainan, Taiwan; 16 Department of Emergency Medicine, Kuang-Tien General Hospital, Taichung, Taiwan; 17 Department of Nursing, Hungkuang University, Taichung, Taiwan; “Mario Negri” Institute for Pharmacological Research, Italy

## Abstract

**Background:**

Carbon monoxide poisoning (COP) often produces severe complications and can be fatal. Because this topic has not been well delineated, we investigated long-term prognoses of patients with COP (COP^[+]^).

**Methods:**

In this retrospective nationwide cohort study, 441 COP^[+]^ patients and 8820 COP^[−]^ controls (120) from 1999 to 2010 were selected from Taiwan’s National Health Insurance Research Database.

**Results:**

Thirty-seven (8.39%) COP^[+]^ patients and 142 (1.61%) controls died (P<0.0001) during follow-up. Incidence rate ratios (IRR) of death were 5.24 times higher in COP^[+]^ patients than in controls (P<0.0001). The risk of death was particularly high in the first month after COP (IRR: 308.78; 95% confidence interval [CI]: 40.79–2337.56), 1 to 6 months after (IRR: 18.92; 95% CI: 7.69–46.56), and 6–12 months after (IRR: 4.73; 95% CI: 1.02–21.90). After adjusting for age, gender, and selected comorbidities, the hazard ratio of death for COP^[+]^ patients was still 4.097 times higher than for controls. Moreover, older age (≥30 years old), male gender, diabetes mellitus, hypertension, and low income were also independent mortality predictors.

**Conclusions:**

COP significantly increases the risk for long-term mortality. Early follow-up and secondary prevention of death are needed for patients with COP.

## Introduction

Carbon monoxide poisoning (COP) is common in the United States; it accounts for an estimated 50,000 emergency department visits annually [1]. COP is the second leading cause of unintentional poisoning deaths and, together with intentional exposures, annually contributes to approximately 2700 fatalities [2], [3].

A diagnosis of COP requires recent CO exposure, symptoms consistent with COP, and an elevated COHb level [4]. Symptoms are required for diagnosis, but no single symptom of COP is sensitive or specific enough to warrant a definitive diagnosis of COP. The most common symptoms are headache, dizziness, nausea/vomiting, confusion, fatigue, chest pain, shortness of breath, and loss of consciousness [4]. Particularly during cold weather, physicians should suspect COP in patients with acute coronary syndrome and arrhythmias. Failing to diagnose COP can have disastrous consequences [4].

Reports of COP-related deaths include only short-term mortality that is clearly related to acute CO toxicity. For example, epidemiologic studies by the USA’s Centers for Disease Control and Prevention [2], [3] use International Statistical Classification of Diseases and Related Health Problems 10th Revision (ICD-10) death certificate codes for cause of death but warn the reader that the “one code specific to CO (T58)” does not distinguish between motor-vehicle exhaust-related deaths and other CO-related deaths [3].

A recent study [5] reported that of the 230 patients they followed up over 8 years, significantly more with COP-induced myocardial injury (n = 32/85) than without (n = 22/145) died. The reason for this increased mortality risk was uncertain. However, this study has two major drawbacks. First, it compared only the COP-related mortality between patients with and without myocardial injury; there was no comparison of mortality between patients with and without COP. Second, it has a selection bias because it enrolled only patients with moderate-to-severe COP treated with hyperbaric oxygen (HBO) at one medical center. A later study [6] showed that adult survivors of acute COP treated with HBO had a higher risk for long-term mortality. The major causes of death with significantly higher mortality rates were mental and psychiatric disorders, injuries, and violence. This study, however, also has a selection bias because it enrolled only patients with COP treated with HBO at one medical center. Findings from the database may not be generalizable to other patients with COP not treated with HBO, other cohorts in the United States, or cohorts in other nations. To determine the long-term prognosis of patients with COP, we thus wanted to analyze a nationwide cohort taken from Taiwan’s National Health Insurance claims database.

## Methods

### Data sources

The Taiwan National Health Insurance (NHI) Program is a universal health care system that covers 99% of the country’s population of 23.3 million [7]. The data used in this analysis were obtained from the National Health Insurance Research Database (NHIRD), which contains all claims data from 1996 through 2011. There were no significant differences in age, gender, or healthcare costs between the sample group and all enrolled residents. The database contains encrypted patient identification numbers, ICD-9-CM (International Classification of Diseases, Ninth Revision, Clinical Modification) codes for applied clinical diagnoses and procedures, details of prescribed drugs, dates of admission and discharge, and basic sociodemographic information, including gender and date of birth. Patients with COP (COP^[+]^) are eligible for free medical care (but not hospital room and board); the expenses of HBO therapy patients are covered by NHI.

### Design

In this longitudinal cohort study, we selected all COP^[+]^ patients who had (1) visited the intensive care unit (ICU) and filed outpatient service claims or (2) filed inpatient service claims between January 1, 1999, and December 31, 2010. Patients were followed from the first reported date of COP to date of death or December 31, 2011, the end of the database period. The controls (20 patients without COP (COP^[−]^) for every COP^[+]^ patient) were randomly selected from the dataset. They were matched with the COP^[+]^ patients by gender, age, and date of index ambulatory care visit or hospitalization.

We linked to the diagnostic codes through the inpatient and outpatient claims databases of the NHI. Our data collection included not only patient survival status, but also date of death, demographics, and baseline comorbidities. Baseline comorbidities, which included diabetes mellitus (DM), hypertension (HTN), coronary artery disease (CAD), stroke, and mental disorder, are important factors affecting mortality and were assessed when the patient was diagnosed with COP. The index date in the COP group is the date that COP was first diagnosed (ICD-9 code 986) in the inpatient database; the index date in the control group was the date assigned based on the index date of the COP^[+]^ group. Other comorbidities that may have presented before the index date were defined as follows: DM (ICD-9 code 250), HTN (ICD-9 codes 401–405), CAD (ICD-9 codes 410–414), stroke (ICD-9 codes 430–438), and mental disorders (ICD-9 codes 290–319). We counted these comorbid conditions if they occurred either in the inpatient setting or in 3 or more ambulatory care claims coded 12 months before the index medical care date. To investigate the association between mortality and COP, only patients with newly diagnosed COP from 1999 to 2010 were included. Follow-ups were done for a minimum of 1 year to determine the incidence of death until the end of 2011. According to the regulations, enrollment in NHI is mandatory for all the population, and must be terminated within 30 days after death. Therefore, patients who were recorded as deceased in the inpatient claim or who had terminated their NHI enrollment within 30 days after discharge from the last hospitalization were presumed dead, and the discharge date was designated as the date of death. This study was conducted according to the Declaration of Helsinki and was approved by the Institutional Review Board at Chi Mei Medical Center. The need for informed consents (written and oral) from the participants was waived because the data set used in this study consists of nationwide, unidentifiable, secondary data released to the public for research. This waiver does not adversely affect the rights and welfare of the patients

### Statistical analysis

The statistical significance of differences in baseline characteristics and comorbid variables between two cohorts was evaluated using Student’s *t* tests for continuous variables and Pearson χ^2^ tests for categorical variables. The risk of death between patients with COP and controls was compared by estimating the incidence rate ratio (IRR) with Poisson regression. Kaplan-Meier analysis was used to calculate the cumulative survival rate between different age groups in the 2 cohorts, and the log-rank test was used to analyze the differences between the survival curves. Thereafter, separate Cox proportional hazard regressions were done to compute the risk of death between the COP and control groups after adjusting for possible confounding factors. SAS for Windows 9.3.1 (SAS Institute, Inc, Cary, NC, USA) was used for this study. Significance was set at P<0.05.

## Results

### Demographic data

After we had excluded ineligible patients, we recruited 441 patients between 1999 and 2010 with COP and 8820 age- and gender-matched controls without COP (COP^[−]^) (Table 1). The mean age in the COP^[+]^ group (36.12±16.25 years) was almost identical to that in the COP^[−]^ group (36.12±16.23). These patients were then subclassified into 3 age groups: 0–29, 30–49, and ≥50. Pearson χ^2^ tests revealed that COP^[+]^ patients were more likely than controls to have comorbid DM, HTN, stroke, and mental disorder (Table 1).

**Table 1:** Demographic characteristics and comorbidities for patients with COP (COP^[+]^) and controls (COP^[−]^).

	COP	Controls	
Characteristic	(n = 441)	(n = 8820)	*P*
Age at index date (years)	36.12±16.25	36.12±16.23	>0.999
[mean ± SD]			
0–29	172 (39.00)	3440 (39.00)	>0.999
30–49	197 (44.67)	3940 (44.67)	>0.999
≥50	72 (16.33)	1440 (16.33)	>0.999
Gender			
Male	226 (51.25)	4520 (51.25)	>0.999
Female	215 (48.75)	4300 (48.75)	>0.999
Baseline comorbidity			
DM			
Yes	28 (6.35)	257 (2.91)	<0.001
No	413 (93.65)	8563 (97.09)	
CAD			
Yes	19 (4.31)	109 (1.24)	<0.001
No	422 (95.69)	8711 (98.76)	
Stroke			
Yes	13 (2.95)	80 (0.91)	<0.001
No	428 (97.05)	8740 (99.09)	
HTN			
Yes	44 (9.98)	519 (5.88)	<0.001
No	397 (90.02)	8301 (94.12)	
Mental disorder			
Yes	185 (41.95)	332 (3.76)	<0.001
No	256 (58.05)	8488 (96.24)	
Geographic region			
North	235 (53.29)	4646 (52.68)	0.004
Center	103 (23.36)	1589 (18.02)	
South	96 (21.09)	2428 (27.53)	
East	10 (2.27)	157 (1.78)	
Monthly income			
< NT$15,840	215 (48.75)	3059 (34.68)	<0.001
NT$15,841–25,000	149 (33.79)	3009 (34.12)	
> NT$25,001	77 (17.46)	2752 (31.20)	

Data are n (%) unless otherwise indicated.

COP, carbon monoxide poisoning; DM, diabetes mellitus; CAD, coronary artery disease; HTN, hypertension; NT$, New Taiwan Dollars.

### Mortality incidence rates

**Figure 1:**
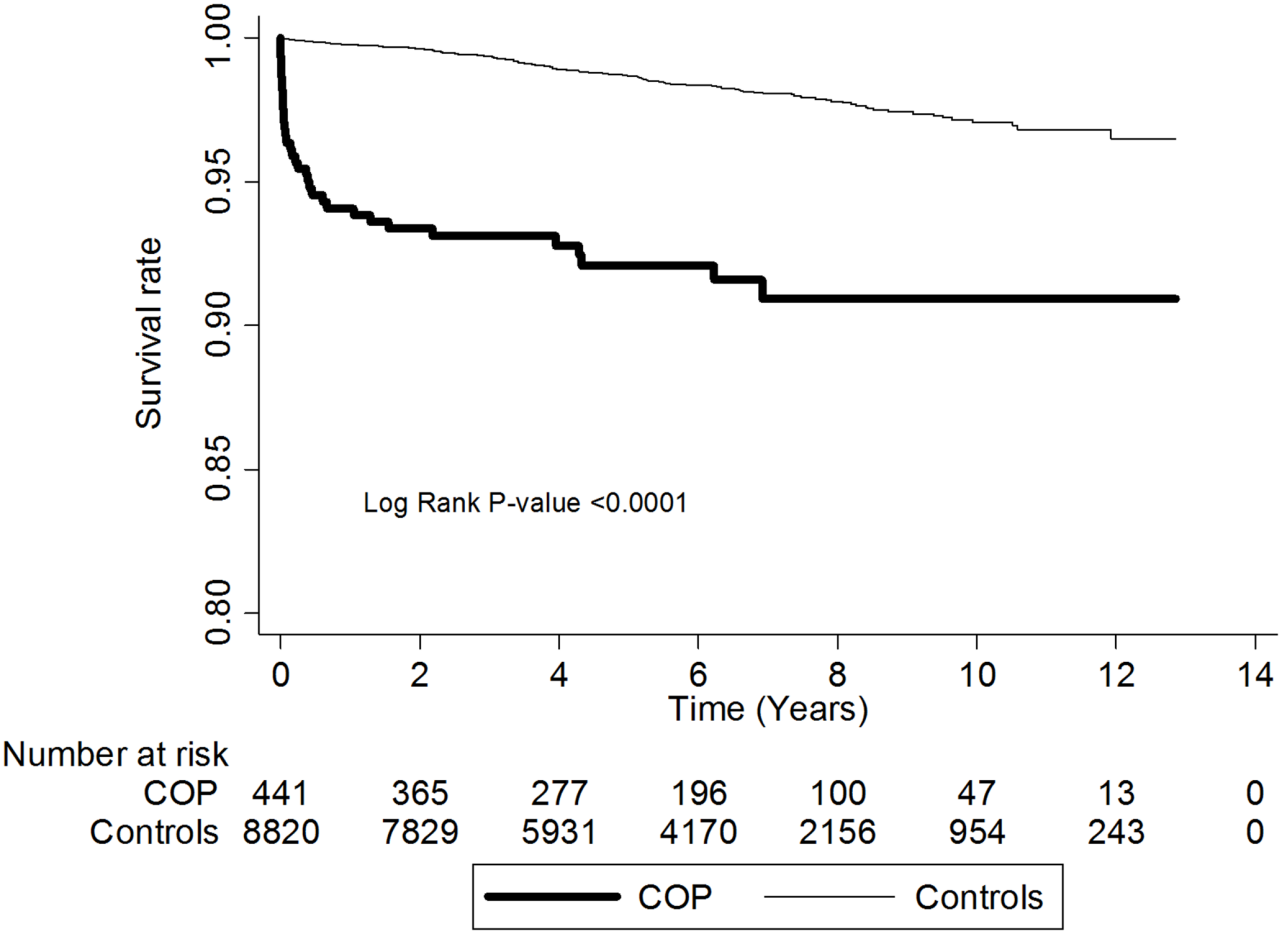
Survival rate for patients with carbon monoxide poisoning (COP^[+]^) and controls (COP^[−]^) during the follow-up.

Of the 9261 patients recruited, 179 (1.93%) died during the follow-up period: 37/441 (8.39%) in the COP^[+]^ group and 142/8820 (1.61%) in the COP^[−]^ group (Table 2). The mortality incidence rates were significantly different: 15.10 (COP^[+]^ group) and 2.72 (COP^[−]^ group) per 1000 person-years (PY) (IRR = 5.24; 95% confidence interval [CI] = 3.62–7.59) (Table 2). The mortality risk was particularly high in the first month after COP (IRR: 308.78; 95% CI: 40.79–2337.56), 1 to 6 months after (IRR: 18.92; 95% CI: 7.69–46.56), and 6–12 months after (IRR: 4.73; 95% CI: 1.02–21.90). Thereafter, the incidence rate was similar in both groups (Table 2). Kaplan-Meier survival analyses and log-rank tests showed that COP^[+]^ patients had a significantly (P<0.0001) lower survival rate than did COP^[−]^ patients during the follow-up period, especially in the first 12 months after COP (Figure 1).

**Table 2:** Risk of death for patients with COP (COP^[+]^) and controls (COP^[−]^).

	COP^[+]^				COP^[−]^					
Characteristics	n	Deaths	PY	Rate	n	Deaths	PY	Rate	IRR (95% CI)	*P*
All	441	37	2450.73	15.10	8820	142	52117.81	2.72	5.24 (3.62–7.59)	<0.001
Age (years)										
0–29	172	7	1105.50	6.33	3440	8	23021.77	0.35	15.62 (5.42–45.01)	<0.001
30–49	197	13	989.34	13.14	3940	24	21050.11	1.14	10.64 (5.32–21.27)	<0.001
≥50	72	17	355.89	47.77	1440	110	8045.93	13.67	3.49 (2.10–5.82)	<0.001
Gender										
Male	226	20	1192.17	16.78	4520	104	25243.40	4.12	4.07 (2.52–6.57)	<0.001
Female	215	17	1258.56	13.51	4300	38	26874.41	1.41	8.43 (4.64–15.32)	<0.001
Comorbidity										
DM	28	7	101.49	68.97	257	34	1403.24	24.23	2.85 (1.26–6.42)	0.012
CAD	19	3	100.16	29.95	109	15	613.56	24.45	1.23 (0.35–4.23)	0.748
Stroke	13	1	66.12	15.12	80	18	381.99	47.12	0.32 (0.04–2.40)	0.269
HTN	44	7	185.42	37.75	519	55	2769.39	19.86	1.90 (0.87–4.17)	0.110
Mental disorder	185	16	882.61	18.13	332	15	1841.92	8.14	2.23 (1.10–4.50)	0.026
Follow-up period										
0–1 month	441	17	35.19	483.09	8820	1	724.38	1.38	308.78 (40.79–2337.56)	<0.001
1–6 months	424	9	171.80	52.39	8819	10	3611.84	2.77	18.92(7.69–46.56)	<0.001
6–12 months	415	2	206.76	9.67	8809	9	4402.11	2.04	4.73(1.02–21.90)	0.047
≥1 year	413	9	2036.98	4.42	8800	122	43379.48	2.81	1.57(0.80–3.09)	0.191

PY, person-years; Rate, mortality rate per 1000 person-years; COP, carbon monoxide poisoning; IRR, incidence rate ratio; DM, diabetes mellitus; CAD, coronary artery disease; HTN, hypertension.

All COP^[+]^ group age subgroups had a higher IRR for death than did their COP^[−]^ group counterparts (Table 2). COP^[+]^ patients 0–29 years old had the highest IRR, then patients 30–49 years old, and then patients ≥50 years old.

Male and female COP^[+]^ patients had significantly higher mortality rates than did male and female COP^[−]^ patients (males: IRR: 4.07, 95% CI: 2.52–6.57, 16.78/1000 PY, 4.12/1000 PY; females: IRR: 8.43; 95% CI: 4.64–15.32) (Table 2).

COP^[+]^ patients with comorbid DM (2.85/1000 PY), and those with a comorbid mental disorder (2.23/1000 PY) had a higher IRR for death than did COP^[−]^ patients comorbid with the same diseases (Table 2). However, COP^[+]^ patients with comorbid CAD, stroke, or HTN did not.

Cox proportional hazard regressions were used to determine crude and adjusted hazard ratios (HRs) for death by cohort for the total sample during the follow-up. After adjusting for patient age, gender, and selected comorbidities, COP (HR: 4.097; 95% CI: 2.736–6.134) was still an independent risk factor for death in the total sample (Table 3). Other risk factors for death included older age (≥30 years old), male gender, DM, HTN, and low income (≤ NT$25,000/mo).

**Table 3:** Crude and adjusted hazard ratios of Cox proportional hazard regressions and 95% confidence interval for death during the follow-up period for study cohort.

	Crude hazard ratio	Adjusted hazard ratio
Cohort	(95% CI)	(95% CI)
COP		
Yes	5.51 (3.84–7.92)*	4.10 (2.74–6.13)*
No	1.000	1.000
Age (years)		
0–29	1.000	1.000
30–49	2.71 (1.48–4.94)*	2.69 (1.47–4.93)*
≥50	24.20 (14.20–41.42)*	12.78 (7.26–22.48)*
Gender		
Male	2.39 (1.73–3.28)*	1.84 (1.34–2.54)*
Female	1.000	1.000
DM		
Yes	10.39 (7.33–14.73)*	2.25 (1.52–3.32)*
No	1.000	1.000
CAD		
Yes	8.37 (5.14–13.62)*	1.12 (0.67–1.90)
No	1.000	1.000
Stroke		
Yes	0.07 (0.04–0.11)*	0.64 (0.38–1.07)
No	1.000	1.000
HTN		
Yes	9.21 (6.77–12.54)*	1.69 (1.16–2.46)*
No	1.000	1.000
Mental disorder		
Yes	3.96 (2.68–5.83)*	1.31 (0.85–2.01)
No	1.000	1.000
Geographic region		
North	0.74 (0.27–2.03)	0.87 (0.32–2.38)
Center	0.77 (0.27–2.17)	0.75 (0.26–2.13)
South	0.87 (0.31–2.39)	0.84 (0.30–2.33)
East	1.000	1.000
Monthly income		
< NT$ 15840	9.18 (4.81–17.53)*	4.53 (2.33–8.78)*
NT$ 15841–25000	5.14 (2.62–10.06)*	3.86 (1.95–7.62)*
> NT$ 25001	1.000	1.000

*P<0.05. CI, confidence interval; COP, carbon monoxide poisoning; DM, diabetes mellitus; CAD, coronary artery disease; HTN, hypertension; NT$, New Taiwan Dollars.

## Discussion

Our study analyzed follow-up claim data on 441 COP^[+]^ patients and 8820 control COP^[−]^ patients. We found that COP raised the risk of long-term mortality and that the mortality incidence was particularly high during the 12 months immediately after acute COP. The risk of death was 308.78 times higher in the COP^[+]^ group than in the COP^[−]^ control group during the first 1 month after COP, 18.92 times higher the first 6 months after, and 4.73 times higher the second 6 months after. After adjusting for potential confounding factors, the risk of death associated with COP^[+]^ patients was still four times higher than that of COP^[−]^ patients. Early referral of COP^[+]^ patients for neurological evaluation, rehabilitation, and secondary death prevention, e.g., by controlling DM and HTN, is important. Socioeconomic assistance may also be urgently needed. To the best of our knowledge, this study is the first nationwide population-based study to evaluate the association between COP and its long-term prognosis.

Older age is an independent predictor of death in the total sample; however, younger patients had a higher IRR for death than did older patients (0–29 years: 15.62 vs. 30–49 years: 10.64 vs. ≥50 years: 3.49) (Table 2). One study [8] showed that older COP^[+]^ patients have a higher risk for cognitive sequelae, but it provided no long-term prognosis about age. It is possibly analogous to the risk for worse outcomes in older patients with traumatic brain injury [8]. Possible age-related mechanisms that affect recovery after brain injury include apoptosis and, for older patients with a closed head injury, carrying the apolipoprotein epsilon 4 genotype [8]. Younger patients may have fewer medical comorbidities; therefore, COP is one of the most likely factors that leads to death.

Comorbid DM and HTN also predict death, which is consistent with a study on myocardial injury and long-term mortality [5]. Although there is no report about whether DM or HTN predisposes a person to COP, COP is a potential cause for developing subsequent DM [9]. COP can cause damage that affects cellular functioning throughout the brain and body: it deprives cells of oxygen and is toxic. The brain and the nervous and endocrine systems are especially sensitive to oxygen deprivation and exposure to toxins [9]. The brain and endocrine system are tightly linked together. Because COP starves the entire brain of oxygen and is toxic, it can damage a wide range of brain functions. If the brain and endocrine system malfunction, then the endocrine system will likely have problems producing or secreting the appropriate hormones; therefore, DM may develop [10].

COP^[+]^ patients with a comorbid mental disorder had a higher IRR for death than did controls comorbid with the same mental disorder. However, a mental disorder was not an independent risk factor for death. There is no published report about whether mental disorders will predispose a person to COP. However, COP is a potential cause for developing a subsequent delayed neuropsychiatric disorder [11].

Low income was also an independent risk factor for death. Although there is no published report about whether low income predisposes a person to COP, one study [12] showed that the death rates from fires, burns, and COP are higher in U.S. households with relatively low incomes. In addition, using carbon monoxide detectors may prevent unintentional COP deaths. Carbon monoxide detector use is lowest in U.S. households with low incomes [13].

There are several limitations to this study. First, the comorbidities relied on the claim data and ICD-9-CM diagnosis codes, which may have resulted in disease misclassification. Second, the medical history of the sampled patients could be traced only back to the year 1999. We cannot be certain that the controls had no COP before January 1999. This could compromise our findings. Third, some important sociodemographic characteristics, such as education level, stress level, body mass index, and alcohol drinking habits; results of clinical examinations; and laboratory data such as COHb; treatment such as HBO therapy; and cause of death were not available in the NHIRD. Therefore, we could not adjust these variables as contributing factors in this study. Fourth, despite our database being national, the findings may not be generalizable to cohorts in other nations.

In summary, our study shows that COP^[+]^ patients had a significantly higher risk of death than did COP^[−]^ controls, especially during the 12 months immediately after an acute COP event. Early follow-up and secondary prevention of death are needed for patients with COP.
